# Association of Depression and Anxiety With the 10-Year Risk of Cardiovascular Mortality in a Primary Care Population of Latvia Using the SCORE System

**DOI:** 10.3389/fpsyt.2018.00276

**Published:** 2018-06-26

**Authors:** Rolands Ivanovs, Anda Kivite, Douglas Ziedonis, Iveta Mintale, Jelena Vrublevska, Elmars Rancans

**Affiliations:** ^1^Department of Psychiatry and Narcology, Riga Stradins University, Riga, Latvia; ^2^Department of Public Health and Epidemiology, Riga Stradins University, Riga, Latvia; ^3^Department of Psychiatry, University of California, San Diego, San Diego, CA, United States; ^4^Department of Cardiology, University Clinic of Paul Stradins, Riga, Latvia

**Keywords:** depressive symptoms, depression, anxiety, anxiety disorders, 10-year cardiovascular mortality risk, SCORE, Latvia

## Abstract

**Background:** Depression and anxiety have been recognized as independent risk factors for both the development and prognosis of cardiovascular (CV) diseases (CVD). The Systematic Coronary Risk Evaluation (SCORE) function measures the 10-year risk of a fatal CVD and is a crucial tool for guiding CV patient management. This study is the first in Latvia to investigate the association of depression and anxiety with the 10-year CV mortality risk in a primary care population.

**Methods:** This cross-sectional study was conducted at 24 primary care facilities. During a 1-week period in 2015, all consecutive adult patients were invited to complete a nine-item Patient Health Questionnaire (PHQ-9) and a seven-item Generalized Anxiety Disorder scale (GAD-7) followed by sociodemographic questionnaire and physical measurements. The diagnostic Mini International Neuropsychiatric Interview (M.I.N.I.) was administered by telephone in the period of 2 weeks after the first contact at the primary care facility. A hierarchical multivariate analysis was performed.

**Results:** The study population consisted of 1,569 subjects. Depressive symptoms (PHQ-9 ≥10) were associated with a 1.57 (95% confidence interval (CI): 1.06–2.33) times higher odds of a very high CV mortality risk (SCORE ≥10%), but current anxiety disorder (M.I.N.I.) reduced the CV mortality risk with an odds ratio of 0.58 (95% CI: 0.38–0.90).

**Conclusions:** Our findings suggest that individuals with SCORE ≥10% should be screened and treated for depression to potentially delay the development and improve the prognosis of CVD. Anxiety could possibly have a protective influence on CV prognosis.

## Introduction

Depression and cardiovascular (CV) diseases (CVD) are the two most common non-communicable diseases causing disability and mortality worldwide (1, 2). In Latvia, the mortality rate from CVD is among the highest in the European Union (EU) and was responsible for 57% of all deaths in 2015 (3, 4). The standard premature mortality from CVD is three times higher in Latvia than in the EU (5).

During the last two decades there is a growing interest in non-conventional cardiovascular risk factors such as psychosocial factors. The seminal INTERHEART study, which considered 15,152 myocardial infarction (MI) cases from 52 countries, showed that nine risk factors were responsible for 90% of the population attributable risk (PAR) and that psychosocial factors (including anxiety and depression) alone were responsible for 32.5% of it, suggesting that they were as important as smoking (PAR, 35.7%) and even more relevant than hypertension (PAR, 17.9%) and diabetes mellitus (PAR, 9.9%) as risk factors for CVD (6). Depression and anxiety have been established as independent risk factors for both the onset of and the prognosis of CVD (7, 8), although the contribution of anxiety as an etiological risk factor is contradictory (9, 10).

Depressive and anxiety disorders are the most frequent mental conditions in patients suffering from CVD and are highly comorbid (11, 12). Between 15 and 20% of CV patients meet the diagnostic criteria for major depressive disorder (MDD), and an even larger proportion (40–65%) indicate subsyndromal levels of depressive symptoms (13, 14). The prevalence of elevated symptoms of anxiety in CV patients has been reported to be approximately 30% (12, 14). Data on the relationship between depression, anxiety and CVD from the Baltic region are limited and controversial (15–17).

The American Heart Association (AHA) has taken a lead role in highlighting the importance of depression in CV patients by recommending routine screening of all cardiac patients in 2008 (18, 19). However, a recent systematic review by Thombs et al. (20) found no evidence that this strategy improves depression or cardiac outcomes (20). Furthermore, in patients with already diagnosed coronary artery disease (CAD), mental health interventions for MDD showed moderate efficacy for reducing cardiac events, but remained controversial in the ability to reduce total mortality (7, 21). This finding underscores the importance of primary prevention of CVD and the need to identify the target population who would most benefit from depression and anxiety screening.

The Systematic Coronary Risk Evaluation (SCORE) function measures the 10-year risk of a fatal CVD. The SCORE charts have been elaborated to rapidly calculate CV mortality risk with sufficient accuracy in both high- and low-risk European populations. Since 1994, the SCORE has been widely advocated by the joint recommendations from the European Association of Preventive Cardiology, European Society of Cardiology, European Atherosclerosis Society and European Society of Hypertension (7, 22). There is also an electronic version of the SCORE risk charts that is even easier to use in daily clinical practice (23).

This project aimed to be the first study to examine the association of anxiety and depression with the 10-year CV mortality risk in a primary care population of Latvia. This research addresses a relevant gap in the body of knowledge concerning Latvian, Baltic and East European populations. The study findings have implications for medical education, policy, and program development in these countries.

## Materials and methods

The current research was performed in 2015 as a part of the National Research Program BIOMEDICINE (2014–2017) to estimate the frequency of most common psychiatric disorders in primary care population of Latvia. The study approached patients of 24 primary care facilities across the country (8 in rural and 16 in urban regions). This research was carried out in the two most widely spoken languages in Latvia (Latvian and Russian).

### Ethics

This study was authorized by the Ethics Committee of the Riga Stradins University, Riga, Latvia (No. 8/18.06.2015.). All subjects were included in the study only after signing written informed consent. The research was conducted accordingly with the Declaration of Helsinki and its subsequent amendments.

### Subjects and procedures

The inclusion criteria were as follows: all consecutive treatment-seeking patients attending primary care facility, persons who were 18 years of age or older, and subjects who had provided their informed consent. The exclusion criteria were as follows: persons who declined to be enrolled in this research project, persons who were younger than 18 years of age, and persons who presented with an urgent health complaints requiring immediate intervention.

During 1 week period in 2015, all consecutive adult persons who corresponded to the inclusion criteria were approached to complete the Patient Health Questionnaire (PHQ-9) for screening of depressive symptoms and the Generalized Anxiety Disorder questionnaire (GAD-7) for screening of anxiety symptoms in Latvian or Russian (language as preferred by participant), followed by a structured interview on sociodemographic characteristics and measurements of weight, height, waist circumference, total cholesterol, and blood pressure. The diagnostic Mini International Neuropsychiatric Interview (M.I.N.I.) was administered by telephone in the period of 2 weeks after the first contact at the primary care facility by specially trained interviewers (psychiatrists). SCORE was calculated for each individual patient using the electronic version of the high risk chart developed and supported by the European Society of Cardiology (23). Information about the prescription of cardiovascular and psychotropic medications from the last 3 months were acquired using medical records.

### Assessment tools and measures

The Patient Health Questionnaire-9 is a nine-item self-reported depression screening tool based on the diagnostic criteria for MDD according to the Diagnostic and Statistical Manual of Mental Disorders, Fourth Edition (DSM-IV) (24). Recent meta-analysis by Meader and colleagues that aimed to define the most effective tool for detection of MDD in persons with chronic somatic diseases showed that among other widely used screening instruments the PHQ-9 revealed good diagnostic accuracy. A score of 10 or higher on the PHQ-9 had a sensitivity of 84% and a specificity of 88% for detecting MDD (25). Both Latvian and Russian versions of the PHQ-9 for Latvia were validated as part of the National Research Project BIOMEDICINE (2014–2017) (26).

The 7-item Generalized Anxiety Disorder scale is a self-rating screening tool for anxiety symptoms (27). The GAD-7 has been established as a reliable tool for detection of most common anxiety disorders. A cut-off score of 10 points and higher has a sensitivity of 89% and a specificity of 82% for generalized anxiety disorder (GAD). It is moderately good at screening panic disorder (PD) (sensitivity-74%, specificity-81%), social anxiety disorder (sensitivity-72%, specificity-80%), and post-traumatic stress disorder (PTSD) (sensitivity-66%, specificity-81%) (28).

The M.I.N.I. is a structured diagnostic interview for most common mental disorders in accordance with the DSM-IV and the 10th version of the International Classification of Diseases (29). The M.I.N.I. has been previously translated for use in Latvian and Russian languages by authorship holders. The M.I.N.I. authorship holder prof. David Sheehan provided written permission to use this scale in our study.

The SCORE system measures the 10-year risk of a fatal CV event (e.g., stroke, myocardial infarction or aneurysm of the aorta). The SCORE risk assessment is derived using data from 12 European cohort studies with 205,178 participants covering a wide geographic spread of countries at different levels of CV risk. The SCORE charts have been elaborated to calculate risk in both high- and low-risk European populations. The reported predictive values representing areas under receiver operating characteristic curves for SCORE have ranged from 0.71 to 0.84 (22). Total CV risk estimation using SCORE is a crucial tool for supporting clinicians during the optimization of individual CV risk reduction in apparently healthy individuals. This risk estimation is based on the following risk factors: gender, age, smoking, systolic blood pressure, and total cholesterol. The threshold for very high risk is defined as a calculated SCORE ≥10%, and it was used as a cut-off score in our study (7, 23).

The sociodemographic questionnaire included questions about demographics (age, sex, ethnicity, marital status, education), CV risk factors, history of CVD, diabetes mellitus and psychiatric disorders, use of CV and psychotropic medications. The section on CV risk factors included questions about family history of premature CVD in first-degree relatives (<55 years in men and <65 years in women), physical activity (number of moderate exercise sessions of at least 30 min per week), smoking habits (smoking status, number of cigarettes), alcohol use, and consumption of fresh vegetables, fruits and fish.

### Statistical analysis

Statistical analysis was performed using Statistical Package for the Social Sciences (SPSS) version 20.0 (IBM SPSS Corp.). Statistical significance was considered as *p* < 0.05. Differences of the study sample between comparative SCORE groups were detected using Chi-Square test or Fisher's exact test.

To examine associations of depression and anxiety with the SCORE function, we conducted an univariate and stepwise multivariate analysis (using binary logistic regression) according to the conceptual hierarchical framework model (30), in which possible confounding variables were distributed into 3 groups: proximal (positive family history, number of cigarettes, diabetes, use of antihypertensive, cholesterol lowering medications), intermediate (body mass index/waist circumference, depression and/or anxiety, sedentary lifestyle, consumption of fresh vegetables and fruits, consumption of fish, alcohol use) and distal factors (education, employment status, marital status, place of residence). Factors that were used in the calculation of the SCORE function (gender, age, blood pressure, total cholesterol, smoking status) were excluded from the list of independent variables in the regression analysis.

As current depressive episode according to the M.I.N.I. and anxiety symptoms detected by the GAD-7 did not show statistically significant results in the univariate analysis, these measures were not included in the final regression model. To gain more statistical power, we combined all anxiety disorders according to the M.I.N.I. into one current anxiety variable for the final analysis, including GAD, PD, PTSD, and agoraphobia. Previous research has demonstrated that these anxiety disorders contribute to the risk of developing CVD and a worse prognosis of CVD (7, 31).

## Results

From 1,756 approached subjects 152 declined to participate in this study. The mean response rate was 91.3%, it varied between 86.3 and 93.7% across 24 primary care facilities all over the country. Those who declined did not significantly differ in the basic sociodemographic characteristics from the study sample. In total, 1,604 patients were approached to complete the PHQ-9 and the GAD-7 questionnaires, which were completed by 1,585 of participants. Among those who completed both questionnaires, the SCORE measure was calculated for 1,569 subjects (69.0% women), who were included in the final analysis. Of the eligible study subjects, 23.4% (*n* = 367) showed a very high 10-year CV mortality risk according to the SCORE (≥10%). Clinical symptoms of depression (PHQ-9 ≥10) were present in 15.0% (*n* = 233) of individuals. According to the M.I.N.I., 10.2% (*n* = 148) had current and 28.1% (*n* = 410) had a lifetime depressive episode. Clinically relevant anxiety symptoms (GAD-7 ≥10) were detected in 10.1% (*n* = 156) of individuals. According to the M.I.N.I., 15.9% (*n* = 232) had a current anxiety disorder. A complete description of the sample is shown in Table 1.

**Table 1:** Description of the study sample.

**Independent variable**	**SCORE** ≥ **10%**		**SCORE** ≤ **9%**		***p***	**Total**	
	***n***	**%**	***n***	**%**		***n***	**%**
**PROXIMAL FACTORS**							
**Positive family history of previous cardiovascular disease**							
Don‘ t know	24	6.6	40	3.3	0.01	64	4.1
Yes	124	33.9	467	39.1		591	37.9
No	218	59.6	688	57.6		906	58.0
**Diabetes mellitus**							
Yes	75	20.4	65	5.6	<0.001	140	9.1
No	292	79.6	1,102	94.4		1,394	90.9
**Antihypertensives**							
Yes	290	79.0	466	38.8	<0.001	756	48.2
No	77	21.0	736	61.2		813	51.8
**Cholesterol lowering medicines**							
Yes	124	33.8	111	9.2	<0.001	235	15.0
No	243	66.2	1,091	90.8		1,334	85.0
**Antidepressants**							
Yes	12	3.3	35	2.9	0.73	47	3.0
No	355	96.7	1167	97.1		1522	97.0
**INTERMEDIATE FACTORS**							
**Body mass index, kg/m**^2^							
<18.50 (underweight)	2	0.5	25	2.1	0.009	27	1.7
18.50–24.99 (normal)	143	24.1	391	30.5		534	34.1
25.00–29.99 (overweight)	132	36.2	419	34.9		551	35.2
30.00+ (obese)	88	39.2	366	32.6		454	29.0
**Waist circumference, cm**							
Increased (88+ females; 102+ males)	209	57.3	557	46.6	<0.001	766	49.1
Normal (≤88 females; ≤102 males)	156	42.7	639	53.4		795	50.9
**Sedentary lifestyle (30 min. of moderate physical activity)**							
Unable to perform	62	17.0	107	9.0	<0.001	169	10.9
1 time a week or less	141	38.7	540	45.4		681	43.8
2–3 times a week	44	12.1	164	13.8		208	13.4
4–6 times a week	35	9.6	139	11.7		174	11.2
Every day	82	22.5	240	20.2		322	20.7
**Depression (PHQ-9)**							
Is present	73	20.2	160	13.4	0.002	233	15.0
Not present	289	79.8	1030	86.6		1319	85.0
**Current depression (M.I.N.I.)**							
Yes	42	12.6	106	9.4	0.09	148	10.2
No	292	87.4	1,017	90.6		1,309	89.8
**Lifetime depression (M.I.N.I.)**							
Yes	97	29.0	313	27.9	0.68	410	28.1
No	237	71.0	810	72.1		1,047	71.9
**Anxiety (M.I.N.I., any anxiety disorder)**							
Is present	43	12.9	189	16.8	0.08	232	15.9
Not present	291	87.1	934	83.2		1,225	84.1
**Anxiety (GAD-7)**							
Yes	37	10.2	119	10.0	0.90	156	10.1
No	325	89.8	1,071	90.0		1,396	89.9
**Generalized anxiety disorder (M.I.N.I.)**							
Yes	18	5.4	73	6.5	0.46	91	6.2
No	317	94.6	1,051	93.5		1,368	93.8
**Panic disorder (M.I.N.I.)**							
Yes	1	0.3	10	0.9	0.47	11	0.8
No	334	99.7	1114	99.1		1459	99.2
**Alcohol use, episodes of heavy drinking in the last year (5 or more doses of alcohol at once)**							
Every day or almost every day	4	1.1	10	0.8	<0.001	14	0.9
3–4 times a week	5	1.4	25	2.1		30	1.9
1–2 times a week	22	6.0	112	9.4		134	8.6
More rare	78	21.3	389	32.6		467	29.9
Never during the past year	257	70.2	659	55.1		916	58.7
**Consumption of fresh vegetables and fruits**							
Yes	258	70.5	896	75.0	0.09	1154	73.9
No	108	29.5	299	25.0		407	26.1
**Consumption of fish**							
Yes	151	41.3	455	38.1	0.28	606	38.8
No	215	58.7	740	61.9		955	61.2
**DISTAL FACTORS**							
**Education**							
9-years basic and unfinished basic education	83	22.7	131	11.0	<0.001	214	13.7
General or vocational secondary and unfinished secondary education	207	56.7	685	57.4		892	57.3
Higher and unfinished higher education	75	20.5	377	31.6		452	29.0
**Employment status**							
Economically inactive	260	71.0	400	33.5	<0.001	660	42.3
Unemployed	16	4.4	72	6.0		88	5.6
Employed	90	24.6	722	60.5		812	52.1
**Marital status**							
Single	19	5.2	134	11.2	<0.001	153	9.8
Live separately, divorced, widowed	144	39.3	310	26.0		454	29.1
Married, cohabiting	203	55.5	750	62.8		953	61.1
**Place of residence**							
Riga	123	33.5	189	15.7	<0.001	312	19.9
Other city	160	43.6	588	48.9		748	47.7
Rural	84	22.9	425	35.4		509	32.4

*SCORE, Systematic Coronary Risk Evaluation, estimates the 10-year risk of a first fatal atherosclerotic event; (22) PHQ-9, a nine-item Patient Health Questionnaire; M.I.N.I., the diagnostic Mini International Neuropsychiatric Interview; GAD-7, a seven-item Generalized Anxiety Disorder scale*.

In the final multivariate analysis model (Table 2), a very high risk of CV mortality was significantly associated with three proximal factors: presence of diabetes, use of antihypertensive and cholesterol lowering medicines. Among distal factors, three variables increased the risk of CV mortality: lower education level, inactive economic status or unemployment, and urban place of residence.

**Table 2:** Factors associated with a very high risk of cardiovascular mortality in univariate and hierarchical multivariate analyses^a^.

**Independent variable**	**OR^b^**	**95% CI**	***p*-Value**	**aOR1^c^**	**95% CI**	***p*-Value**	**aOR2^d^**	**95% CI**	***p*-Value**	**aOR3^e^**	**95% CI**	***p*-Value**
**PROXIMAL FACTORS**												
**Positive family history of early cardiovascular disease**												
Don‘t know vs. no	1.89	1.12–3.21	0.02	1.81	1.01–3.24	0.045	2.09	1.08–4.06	0.03	1.23	0.62–2.46	0.55
Yes vs. no	0.84	0.65–1.08	0.17	0.66	0.50–0.87	0.003	0.69	0.51–0.93	0.01	0.88	0.64–1.20	0.42
**Diabetes mellitus**												
Yes vs. no	4.36	3.05–6.22	<0.001	2.45	1.66–3.62	<0.001	2.88	1.84–4.52	<0.001	2.52	1.63–3.89	<0.001
**Antihypertensive medications**												
Yes vs. no	5.95	4.51–7.85	<0.001	4.26	3.17–5.74	<0.001	5.13	3.59–7.32	<0.001	3.22	2.29–4.53	<0.001
**Cholesterol lowering medications**												
Yes vs. no	5.02	3.75–6.71	<0.001	2.55	1.86–3.51	<0.001	2.50	1.77–3.52	<0.001	2.07	1.44–2.95	<0.001
**Antidepressants**												
Yes vs. no	1.13	0.58–2.20	0.73	1.05	0.50–2.21	0.90						
**INTERMEDIATE FACTORS**												
**Body mass index, kg/m**^2^												
< 18.50 (underweight) vs. 18.50–24.99 (normal)	0.33	0.08–1.43	0.14				0.31	0.04–2.48	0.27			
25.00–29.99 (overweight) vs. 18.50–24.99 (normal)	1.29	0.97–1.78	0.08				0.95	0.65–1.40	0.80			
30,00+ (obese) vs. 18.50–24.99 (normal)	1.52	1.13–2.06	0.006				0.76	0.46–1.25	0.28			
**Waist circumference, cm**												
Increased (88+ females; 102+ males) vs. Normal (≤88 females; ≤102 males)	1.54	1.21–1.98	<0.001				0.71	0.48–1.07	0.10			
**Sedentary lifestyle–30 min of moderate physical activity**												
4–6 times a week vs. Every day	0.74	0.47–1.15	0.18				0.93	0.55–1.56	0.78			
2–3 times a week vs. Every day	0.79	0.52–1.19	0.26				0.88	0.54–1.44	0.61			
1 time a week or less vs. Every day	0.76	0.56–1.04	0.09				0.70	0.48–1.02	0.06			
Unable to perform vs. Every day	1.70	1.14–2.53	0.01				1.04	0.64–1.70	0.87			
**Depression (PHQ-9)**												
Is present vs. not present	1.63	1.18–2.21	0.002				1.65	1.12–2.42	0.01	1.57	1.06–2.33	0.03
**Anxiety (M.I.N.I., any anxiety)**												
Is present vs. not present	0.73	0.51–1.04	0.08				0.58	0.38–0.89	0.01	0.58	0.38–0.90	0.02
Alcohol consumption, episodes of heavy drinking in the last year (5 or more doses of alcohol at once)												
Every day or almost every day vs. Never during the past year	1.03	0.32–3.30	0.97				1.24	0.30–5.14	0.76			
3–4 times a week vs. Never during the past year	0.51	0.19–1.35	0.18				0.68	0.21–2.18	0.51			
1–2 times a week vs. Never during the past year	0.50	0.31–0.81	0.005				0.94	0.51–1.73	0.84			
More rare vs. Never during the past year	0.51	0.39–0.68	<0.001				0.78	0.55–1.10	0.15			
**Consumption of fresh vegetables and fruits (every day)**												
No vs. yes	1.25	0.97–1.63	0.09				1.12	0.81–1.54	0.50			
Consumption of fish (≥2 times a week)												
No vs. yes	0.88	0.69–1.11	0.28				0.96	0.71–1.28	0.77			
**DISTAL FACTORS**												
**Education**												
9-years basic and unfinished basic education vs. Higher and unfinished higher education	3.19	2.20–4.61	<0.001							2.25	1.39–3.65	0.001
General or vocational secondary and unfinished secondary education vs. Higher and unfinished higher education	1.52	1.13–2.04	0.005							1.29	0.90–1.84	0.17
**Employment status**												
Economically inactive vs. Employed	5.21	3.98–6.82	<0.001							2.87	2.07–3.98	<0.001
Unemployed vs. Employed	1.78	0.99–3.20	0.052							2.05	1.05–4.01	0.04
**Marital status**												
Single vs. Married, cohabiting	0.52	0.32–0.87	0.01							0.65	0.35–1.23	0.19
Live separately, divorced, widowed vs. Married, cohabiting	1.72	1.34–2.21	<0.001							0.94	0.69–1.29	0.71
**Place of residence**												
Riga vs. rural	3.29	2.38–4.56	<0.001							4.00	2.62–6.10	<0.001
Other city vs. rural	1.38	1.03–1.84	0.03							1.55	1.08–2.22	0.02

*OR, odds ratio; aOR, adjusted odds ratio; CI, confidence interval; PHQ-9, a nine-item Patient Health Questionnaire; M.I.N.I., the diagnostic Mini International Neuropsychiatric Interview*.

a *Cells in the table are left empty intentionally due to the stepwise multivariate analysis according to conceptual hierarchical framework model (see Methods part for more detailed information)*.

b *OR, crude odds ratio*.

c *aOR1, adjusted odds ratio in first model, proximal factors adjusted*.

d *aOR2, adjusted odds ratio in second model, proximal, and intermediate factors adjusted*.

e *aOR3, adjusted odds ratio in third model, proximal, intermediate, and distal factors adjusted*.

The only intermediate factors that remained significantly associated with a SCORE ≥10% after adjustment for socio-economic and traditional CV risk factors were depression (according to the PHQ-9) and anxiety (according to the M.I.N.I.). Subjects with clinical symptoms of depression had a 1.57 (*p* = 0.03) times higher odds of very high CV risk. Interestingly, current anxiety disorder showed a preventive effect on CV mortality. Subjects with diagnosed anxiety disorder had a 0.58 lower odds (*p* = 0.02) of having a SCORE ≥10%.

## Discussion

This is the first study in Latvia to explore the relationship between depression, anxiety and the 10-year risk of a first fatal atherosclerotic event in primary care population based on the SCORE system. The most relevant findings were that patients with clinical symptoms of depression (PHQ-9 ≥10) demonstrated a 1.57 times higher odds of a very high 10-year CV risk as measured by the SCORE function, but current anxiety disorder (M.I.N.I.) reduced the risk of CV mortality with an OR of 0.58. These findings remained statistically significant even after adjusting for multiple socio-demographic and traditional CV risk factors.

A global overview of the literature from approximately 50 prognostic studies on the link between depression and CVD from the last 25 years concluded that clinically relevant depressive symptoms are associated with a 1.6 to 2.2-fold higher risk of adverse outcomes (31, 32); however, prior studies in the Baltic nations have been contradictory. A prospective cohort study of primary care population (*n* = 1,115) in Lithuania examined the association of the metabolic syndrome, current major depressive episode, lifetime major depressive episode, and GAD with 10-year CV mortality (33). Butnoriene et al. (33) found that lifetime major depressive episode was associated with an elevated risk of CV mortality in women (HR = 1.86; *p* = 0.019) adjusted for conventional CV risk factors. In men, neither current MDE nor lifetime MDE were associated with mortality. Another study from Lithuania by Burokiene et al. (15) showed a more modest but statistically significant association of CV morbidity and clinically relevant depressive symptoms (OR = 1.18; *p* = 0.001). Surprisingly, a cross-sectional study involving 1,094 patients from 23 family practices across Estonia did not indicate higher co-morbidity of CVD in depressed patients when compared to non-depressed patients (16). Our findings about the association of depression with CV mortality are in line with prior studies identifying depression as an independent risk factor for CV morbidity and mortality.

Although many studies have examined the association of depression with separate traditional CV risk factors, including arterial hypertension, hypercholesterolemia, diabetes and obesity, we found only one publication that used the SCORE system (34). Koponen et al. (34) reported that clinically relevant depressive symptoms were associated with a 2.9-fold higher (95% CI 1.4–5.7) 10-year CV mortality risk in men and a 1.4-fold higher (95% CI 1.1–4.2) risk in women using the SCORE function. Despite similar objectives, there were several significant differences in the methodology of this study. In the study by Koponen et al. (34) a “high/very high” risk for CV mortality was defined as a SCORE ≥3%. We chose a SCORE ≥10% as a threshold for very high risk of CV mortality in accordance with the European Guidelines on cardiovascular disease prevention in clinical practice (2016) (7). We also used additional confounding factors such as place of residence and anxiety, which appeared to have a significant impact on the SCORE results.

In contrast to depression, the relationship between anxiety and CVD is less clear. Meta-analysis by Roest et al. (35) summarizing 20 studies with 249,846 participants found that anxious persons were at an increased risk for incident CAD (HR = 1.26; *p* < 0.0001) and CV mortality (HR = 1.48; *p* = 0.003), independent of sociodemographic characteristics, traditional and lifestyle CV risk factors (35). However, the meta-analytic assessment on the link between anxiety and CAD was not controlled for depression, which is very frequent comorbid condition with anxiety. Since the publication of that review in 2010, more recent studies have suggested that anxiety may act as a protective factor in certain instances (9, 10). The most recent meta-analysis (2016) also included studies focusing on stroke and peripheral vascular disease, summarizing 37 studies with 1,565,699 participants (36). Batelaan and colleagues found that clinically relevant anxiety symptoms were associated with a 1.52 times higher risk of incident CV morbidity (95% CI 1.36–1.71). Although the data on the prognostic influence of anxiety are complex and even controversial, most publications support the association of anxiety with CV mortality, but they do so to a lesser extent compared with depression. In addition, a few studies have been performed in the Baltic region, but none have examined the association of anxiety with the SCORE function. Butnoriene et al. (33) showed that current GAD predicted greater CV mortality in women (HR = 1.86–1.99; *p* ≤ 0.025), but not in men. A small study (*n* = 64) from Estonia also reported differences between young male and female post-MI patients, indicating that females suffered a higher level of cognitive worry and were less able to relax in the prodromal period of MI (37).

One of the most unexpected findings of our study was that current anxiety disorder (M.I.N.I.) was associated with a reduced CV mortality risk, suggesting a possible protective influence. This result is in agreement with the findings of Meyer et al. (9), which showed that elevated symptoms of anxiety were associated with beneficial effects on survival in individuals with stable CV conditions (HR = 0.70; *p* = 0.031) comparing to individuals after acute MI with reduced systolic left ventricular function (HR = 1.32; *p* = 0.011) (9). Therefore, it has been hypothesized that prognostic influence of anxiety might be modulated through CVD severity (degree of left ventricular dysfunction).

The use of a structured diagnostic interview for the detection of anxiety disorders, a nationally representative primary care convenience sample including persons with a wide range of ages, and a hierarchical multivariate analysis including control for conventional CV, socioeconomic risk factors, anxiety and place of residence are important strengths of this study.

There are several limitations to the results of current study. First, because of the cross-sectional setting of this study, we could not draw definite conclusions about the causality of the identified relationships between clinically relevant depressive symptoms or anxiety disorders and cardiovascular mortality risk. Second, the PHQ-9 with a cut-off score of 10 points and higher has been accepted as a reliable instrument for detection of MDD in chronic physical diseases (25). However, the PHQ-9 is a screening tool and not a diagnostic criterion for MDD, which can result in false positive cases. Third, we combined all anxiety disorders according to the M.I.N.I., including GAD, PD, agoraphobia, and PTSD, in the current anxiety variable of the final analysis to gain more statistical power. Although these diagnoses share the common basic symptoms of anxiety and neurobiological mechanisms (38, 39), we were unable to clarify whether our findings were attributable to all included anxiety disorders.

## Conclusions

We found a statistically significant association between depression (PHQ-9 ≥10) and a very high risk of CV mortality. In contrast to our expectations, current anxiety disorder (M.I.N.I.) was found to be negatively associated with CV mortality risk (SCORE ≥10%), which suggests that anxiety could potentially have a protective influence on CV prognosis. The findings of this study suggest that individuals with the SCORE ≥10% could benefit from screening and treatment of depression to potentially delay the development and improve the prognosis of CVD. Further research is needed to investigate the influence of anxiety on CV mortality risk in Latvian population.

## Author contributions

ER and JV created the design of this study. ER coordinated the study. DZ consulted about mental health aspects and IM consulted about cardiovascular aspects of the study design and statistical analysis. JV and RI participated in the data collection. AK performed the analyses. RI wrote the first draft of the manuscript. All authors participated in the writing and revision of the successive drafts of the manuscript. All authors read and approved the final manuscript.

### Conflict of interest statement

The authors declare that the research was conducted in the absence of any commercial or financial relationships that could be construed as a potential conflict of interest.
